# Neurofilament Transport Is Bidirectional *In Vivo*

**DOI:** 10.1523/ENEURO.0138-22.2022

**Published:** 2022-08-24

**Authors:** Nicholas P. Boyer, Jean-Pierre Julien, Peter Jung, Anthony Brown

**Affiliations:** 1 Department of Neuroscience, The Ohio State University, Columbus, Ohio 43210; 2 Department of Psychiatry and Neuroscience, CERVO Brain Research Centre, University Laval, Québec City, QC G1J 2G3, Canada; 3 Department of Physics and Astronomy and Quantitative Biology Institute, Ohio University, Athens, Ohio 45701

**Keywords:** axon, axonal transport, green fluorescent protein, neurofilament, photoactivation

## Abstract

Neurofilaments are abundant space-filling cytoskeletal polymers that are transported into and along axons. During postnatal development, these polymers accumulate in myelinated axons causing an expansion of axon caliber, which is necessary for rapid electrical transmission. Studies on cultured nerve cells have shown that axonal neurofilaments move rapidly and intermittently along microtubule tracks in both anterograde and retrograde directions. However, it is unclear whether neurofilament transport is also bidirectional *in vivo*. Here, we describe a pulse-spread fluorescence photoactivation method to address this in peripheral nerves dissected from *hThy1-paGFP-NFM* transgenic mice, which express a photoactivatable fluorescent neurofilament protein. Neurofilaments were photoactivated in short segments of myelinated axons in tibial nerves at 2, 4, 8, and 16 weeks of age. The proximal and distal spread of the fluorescence due to the movement of the fluorescent neurofilaments was measured over time. We show that the directional bias and velocity of neurofilament transport can be calculated from these measurements. The directional bias was ∼60% anterograde and 40% retrograde and did not change significantly with age or distance along the nerve. The net velocity decreased with age and distance along the nerve, which is consistent with previous studies using radioisotopic pulse labeling. This decrease in velocity was caused by a decrease in both anterograde and retrograde movement. Thus, neurofilament transport is bidirectional *in vivo*, with a significant fraction of the filaments moving retrogradely in both juvenile and adult mice.

## Significance Statement

Neurofilaments are important structural components of axons that are transported from the cell body to the axon tip in a “stop-and-go” manner. Here we show that neurofilament transport is bidirectional in mature myelinated axons *in vivo*, with a significant fraction of the filaments moving backward toward the cell body. The fact that axons invest metabolic energy to move neurofilaments backwards as well as forwards in axons is puzzling, and it suggests that neurofilament transport is not simply a mechanism to deliver neurofilaments to axons. We speculate that the bidirectional movement of neurofilaments functions to also distribute and organize these polymers in axons, which is a different way of thinking about axonal transport.

## Introduction

The conduction velocity of axons is dependent on their cross-sectional area, which can increase up to 50-fold during postnatal development in mammals (Waxman, 1980; Nilsson and Berthold, 1988). This axonal expansion is driven in part by an accumulation of neurofilaments, which are space-filling cytoskeletal polymers that move into and along axons by the mechanisms of axonal transport (Hoffman, 1995). In the absence of neurofilaments, axons fail to expand in caliber, resulting in reduced conduction velocities (Ohara et al., 1993; Sakaguchi et al., 1993; Zhu et al., 1997; Kriz et al., 2000; Lancaster et al., 2018). Thus, neurofilaments are an important determinant of one of the basic cable properties of axons.

The axonal transport of neurofilament proteins was discovered using radioisotopic pulse-labeling (Hoffman and Lasek, 1975). Over the years, such experiments have demonstrated that a pulse of radiolabeled, newly synthesized neurofilament proteins forms a spreading Gaussian wave that propagates slowly in an anterograde direction with a modal velocity ranging from 0.1 to 1 mm/d, depending on neuronal cell type and age (Brown, 2014). More recently, live-cell imaging of fluorescently tagged neurofilament proteins has shown that these proteins move in the form of neurofilament polymers (Roy et al., 2000; Wang et al., 2000; Yan and Brown, 2005). The polymers move intermittently along microtubule tracks in a “stop-and-go” manner propelled by dynein and kinesin motors, switching between kinetically distinct on-track and off-track states (Francis et al., 2005; Trivedi et al., 2007; Uchida et al., 2009). Neurofilaments in the on-track state alternate between short bouts of rapid movement and short pauses with a duration on the order of seconds or minutes, whereas neurofilaments in the off-track state pause for an hour or more without movement (Monsma et al., 2014; Walker et al., 2019). Thus, the wave in radioisotopic pulse-labeling experiments is thought to represent a population of radiolabeled neurofilament polymers that move at a broad range of rates dictated by their stochastic, asynchronous, and intermittent movement (Brown et al., 2005; Li et al., 2012).

An additional and surprising finding in the live imaging studies on cultured neurons was that neurofilament movement is also bidirectional, with a significant proportion of the filaments moving retrogradely. For example, in our kymograph analysis of neurofilament transport in rat cortical neurons, the filaments spent 56% of the time moving anterogradely and 44% moving retrogradely (Fenn et al., 2018a). An important question, therefore, is how this bidirectional movement in cultured neurons can be reconciled with the apparently unidirectional anterograde movement observed by radioisotopic pulse-labeling *in vivo*. One possible explanation is that the bidirectional movement is unique to immature unmyelinated axons in culture. Alternatively, neurofilament transport may be bidirectional *in vivo*, albeit with a net anterograde bias. If this were the case, it would raise intriguing questions about why neurons invest energy to move neurofilaments both forward and backward in axons.

Here, we use *hThy1-paGFP-NFM* transgenic mice to establish the directionality of neurofilament transport *in vivo*. These mice express low levels of photoactivatable GFP-tagged neurofilament protein (paGFP-NFM) in neurons, including all myelinated axons of the sciatic and tibial nerves (Walker et al., 2019). Since single neurofilaments cannot be resolved in these neurofilament-rich axons, we adapted our previously described fluorescence photoactivation pulse-escape technique (Trivedi et al., 2007; Li et al., 2014). In this new approach, which we call the pulse-spread technique a population of neurofilaments is marked by activation of paGFP-NFM in an axon, and the spread of the activated fluorescence proximally and distally is analyzed over time (Boyer et al., 2020). We show here that this method permits, for the first time, calculation of the anterograde and retrograde neurofilament flux, directional bias, and average population velocity of neurofilaments in mature myelinated axons of peripheral nerves from juvenile and adult mice using fluorescence microscopy.

## Materials and Methods

### Animals

Mice were housed in a vivarium at The Ohio State University and were maintained and euthanized in accordance with procedures approved by the university Institutional Animal Care and Use Committee. The method of euthanasia was CO_2_ inhalation followed by cervical dislocation. The production, genotyping, and characterization of the *hThy1-paGFP-NFM* mice, which express paGFP-tagged neurofilament protein M under the control of neuron-specific portions of the human Thy1 promoter, were described previously (Walker et al., 2019). The colony was maintained on a C57BL/6J background by crossing mice heterozygous for the transgene with wild-type C57BL/6J background mice (The Jackson Laboratory). The mice bred normally and exhibited no overt phenotype. The transgene exhibited Mendelian inheritance, and all experiments were performed on heterozygotes.

### Nerve dissection and preparation

Tibial and sciatic nerves were dissected and excised as described in detail previously (Boyer et al., 2020). The nerve segments (∼1.5 cm long) were placed immediately into either oxygenated Breuer’s saline (98 mm NaCl, 1 mm KCl, 2 mm KH_2_PO_4_, 1 mm MgSO_4_, 1.5 mm CaCl_2_, 5.6% d-glucose, and 23.8 mm NaHCO_3_) or inhibitor saline (98 nm NaCl, 1 mm KCl, 2 mm KH_2_PO_4_, 1 mm MgSO_4_, 1.5 mm CaCl_2_, 23.8 mm NaHCO_3_, 5.6% 2-deoxy-d-glucose, and 0.5 mm sodium iodoacetate). The oxygenation was performed by bubbling 95% O_2_/5% CO_2_ through the solution for 30 min. The maximum time between death and immersion of the nerve in the saline was 10 min. Once in saline, the nerve sheath was removed gently under a stereomicroscope using fine dissecting forceps and the exposed nerve was placed onto a coverslip and mounted in a closed-bath observation and perfusion chamber (model FCS2, Bioptechs) using either a 100 μm silicone gasket for tibial nerves or a 500 μm silicone gasket for sciatic nerves. The chamber was filled with saline using a syringe, taking care to avoid bubbles, and transferred to the microscope stage where it was connected to a Sage Instruments syringe pump (Thermo Fisher Scientific). Saline flow was maintained at a rate of 0.25 ml/min. Temperature was maintained at 37°C throughout the course of the experiments using built-in heaters in the Bioptechs chamber and by warming the saline using an inline solution heater (Warner Instruments) placed between the syringe pump and the chamber. To avoid loss of heat via the oil-immersion objective, the objective was heated to 37°C using an objective heater (Okolab).

### Pulse-spread imaging

The paGFP-NFM fluorescence was activated and imaged as described in detail previously (Boyer et al., 2020). The nerve preparations were observed using a Revolution WD Spinning Disk Laser Confocal Imaging System (Andor) controlled by MetaMorph software (Molecular Devices). The system included a motorized confocal scanning unit (model CSU-W1, Yokogawa), an inverted epifluorescence microscope (model TiE, Nikon), an epifluorescence light source (SOLA LED, Lumencor), and a black illuminated electron-multiplying CCD camera (model iXon ULTRA 897, Andor). All imaging was performed using a CFI Plan Apo VC 100×/1.4 numerical aperture oil-immersion objective (Nikon). Myelinated axons were located under bright-field illumination. For the best optical quality, we focused on the axons on the bottom surface of the desheathed nerve, immediately adjacent to the glass coverslip. A single confocal image was acquired using 488 nm excitation with 200 s exposure at 25% laser power to reduce the autofluorescence. A 40-μm-wide rectangular region was then drawn extending across the entire field perpendicular to the axons to cross as many axons as possible. A full-field preactivation image was acquired with 4 s exposure and 5% laser power, followed by activation of the paGFP fluorescence in the rectangular region using an laser galvo targeted illumination system (model FRAPPA, Andor) with five pulses of 405 nm light and a 40 μs pixel dwell time. The activation took ∼25 s, resulting in fluorescent labeling of neurofilaments in a 40-μm-long segment of each axon. A postactivation image was acquired immediately, and then a series of time-lapse images were acquired every 30 s for 10 min, beginning 1 min after activation. For the supplemental movies, we extended the duration of the time-lapse acquisition to 30 min. The 1 min delay was necessary to allow for the increase in fluorescence (recovery from the dark state) that is observed following photoactivation of paGFP (Bancaud et al., 2010). We considered the end of this 1 min delay to be the start of our pulse-spread time course (*t* = 0).

### Image processing and analysis

All image processing and analysis was performed using FIJI software (Schindelin et al., 2012). Raw fluorescence images were subjected to flat-field, dark-field, and photobleach correction before analysis, as described previously (Boyer et al., 2020). The flat-field correction image was acquired at the end of each imaging session using a concentrated fluorescein solution as a uniform planar fluorescence source (Model, 2014). The dark-field correction image was obtained by averaging a set of 100 images that were acquired with an exposure time of zero and with all light path shutters closed. The photobleaching rate ɣ was approximated by fitting the fluorescence decay of all glycolytically inhibited axons to an exponential trendline of the form $e^{-t\gamma }$, where *e* is the natural logarithm base, t is the time, and γ is the exponential bleaching rate. The purpose of using glycolytically inhibited axons was to eliminate the contribution of neurofilament transport to the loss of fluorescence, thereby ensuring that it was due entirely to photobleaching.

### Mathematical analysis

**Figure 1.**
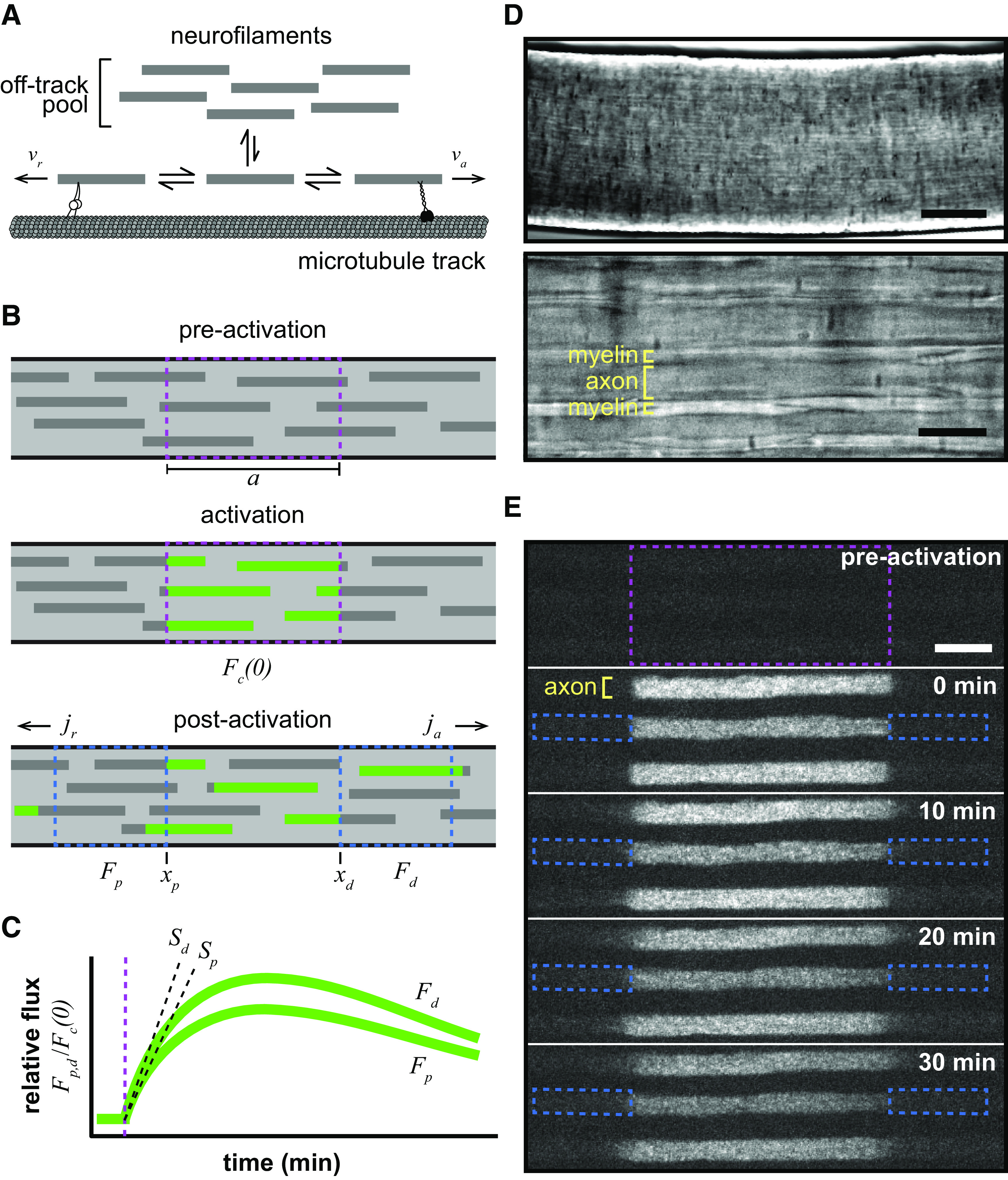
The pulse-spread experimental paradigm. ***A***, Neurofilaments switch between kinetically distinct on-track and off-track pausing states. When on track, the filaments switch between the on-track pausing state and anterograde and retrograde transported states moving at rates of *v_a_* and *v_r_*, respectively. The motors linking the moving neurofilaments to the microtubule track are meant to represent kinesin-1 (anterograde) and dynein (retrograde). ***B***, Diagram of a pulse-spread experiment in which photoactivatable neurofilaments are activated within an axon segment (dashed magenta box), after which the fluorescence (*F_d_* and *F_p_*) is measured within the flanking windows (dashed blue boxes) and neurofilament flux (*j_a_* and *j_r_*) is calculated (see Materials and Methods). ***C***, Graph of the theoretical pulse-spread kinetics, showing the rise and fall of the fluorescence in the distal and proximal flanking windows (*F_d_* and *F_p_*, respectively) with time, normalized to the fluorescence in the central window immediately after photoactivation (*F_c_*(0)). The rate of increase in the fluorescence at short times is given by the initial slopes *S_p_* and *S_d_*. The time of fluorescence activation is marked by the vertical dashed magenta line. ***D***, Bright-field transmitted light microscopy images of the tibial nerve within the perfusion chamber at 10× (top; scale bar, 100 μm) and 100× (bottom; scale bar, 10μm) magnification. Myelinated axons can be distinguished by their myelin sheaths. ***E***, Time-lapse fluorescence images from a pulse-spread experiment in the tibial nerve of an *hThy1-paGFP-NFM* heterozygous male mouse, showing three axons spaced apart by their myelin sheaths. The dashed magenta box marks the activated region, and the dashed blue boxes mark the proximal and distal flanking regions for one axon at *t* = 0 min. Note that there is some loss of fluorescence because of photobleaching in these raw images. Our quantitative analysis was confined to the first 4 min after activation, and the data were corrected for photobleaching (see Materials and Methods). Proximal is left; distal is right. Scale bar, 10 μm.

We describe here how we used the bidirectional spreading of photoactivated neurofilaments reported in this study to extract information about the directionality and velocity of neurofilament transport. The filaments are considered to move bidirectionally and intermittently along microtubule tracks and to switch between running and pausing states, spending most of their time in the pausing states (Brown, 2000). Filaments in the on-track state exhibit short “on-track” pauses, whereas filaments in the “off-track” state disengage from their tracks and pause for prolonged periods (Trivedi et al., 2007; Li et al., 2012; Fig. 1*A*). Denoting the quantity of neurofilaments moving in the anterograde and retrograde directions per unit length of axon by $\rho_{a}$ and $\rho_{r}$ and their corresponding speed by $v_{a}$ and $v_{r}$, the fluxes of transported neurofilament polymer in the anterograde and retrograde directions are given as follows:

$$j_{a}=\rho_{a}v_{a},$$

and

(1) $$j_{r}=-\rho_{r}v_{r},$$

respectively, and the total flux j is given as follows:

(2) $$j=j_{a}+j_{r}=\rho_{a}v_{a}-\rho_{r}v_{r}.$$

Here $\rho_{a}$ and $\rho_{r}$ are reported in micrometers of polymer; $v_{a}$ and $v_{r}$ are reported in micrometers per second; and   j, $j_{a}$, and $j_{r}$ are reported in seconds. Since the flux at a given location along the axon is the quantity of neurofilament polymer that moves past that location in a unit of time, it is related to the average neurofilament velocity $\bar{v}$ (averaged over all neurofilaments at that location, on-track and off-track) through $j=\rho \bar{v}$, where ρ denotes the quantity of all neurofilaments (moving and pausing) per unit length of axon. Thus, we can write the following:

(3) $$\bar{v}=\frac{\rho_{a}}{\rho }v_{a}-\frac{\rho_{r}}{\rho }v_{r}=p_{a}v_{a}-p_{r}v_{r},$$

where $p_{a}$ and $p_{r}$ are the fractions of all neurofilaments in the anterograde and retrograde moving states. If the flux $j_{a}$ everywhere along the axon matches the net flux of neurofilaments entering the axon $j_{in}$, then the kinetics of neurofilaments switching between moving and pausing states is in steady state. In other words, the quantities $\rho_{a}$and $\rho_{r}$, the fractions $p_{a}$ and $p_{r}$, and the fluxes $j_{a}$ and $j_{r}$ assume the time-independent values $\rho_{a}^{s}\text{, }\rho_{r}^{s}\text{, }p_{a}^{s}\text{, }p_{r}^{s}\text{, }j_{a}^{s},\text{ and }j_{r}^{s},$ respectively, and the velocity in Equation 3 is the steady-state average velocity of the neurofilaments.

In a pulse-spread experiment, we initially ( t=0) activate the fluorescence of a population of axonal neurofilaments within a central activation window of length a (Fig. 1*B*). We then track this labeled population over time *t* to determine the amount of fluorescence remaining in the central window [i.e., $F_{c}(t)$], and the amounts of fluorescence in the proximal and distal flanking windows [i.e., $F_{p}(t)$ and $F_{d}(t)$; Fig. 1*C*]. The amount of neurofilament in the central window declines by the sum of the neurofilament fluxes $j_{a}$ and $j_{r}$ due to neurofilaments leaving the central window at its distal and proximal ends $x_{d}$ and $x_{p}$ according to the following equation of continuity:

(4) $$\frac{dF_{c}}{dt}=-j_{a}(x_{d},t)+j_{r}(x_{p},t)=-\left(\rho_{a}(x_{d},t)v_{a}+\rho_{r}(x_{p},t)v_{r}\right),$$

where $\rho_{a}(x_{d},t)$ and $\rho_{r}(x_{p},t)$ denote the quantity of labeled neurofilaments in the anterograde and retrograde moving states at the distal and proximal ends of the central window, respectively. The initial quantity of activated neurofilaments in the central window is given by $F_{c}(0)=\rho a$, where a is the length of the central window. Normalizing the time-dependent content $F_{c}(t)$ by the initial content [i.e., ${\tilde{F}}_{c}(t)=F_{c}(t)/F_{c}(0)$], this rate of loss then becomes the following:

(5) $$\frac{d{\tilde{F}}_{c}}{dt}=-\frac{v_{a}p_{a}(x_{d},t)+v_{r}p_{r}(x_{p,}t)}{a},$$

where $p_{a}(x_{d},t)$ and $p_{r}(x_{p},t)$ denote the fractions of labeled neurofilaments in the anterograde and retrograde moving states at the distal and proximal ends of the central window, respectively. These fractions decline with time as the moving neurofilaments leave the central window. At early times after activation, Taylor expansion of this differential equation reveals a linear decay, as follows:

(6) $${\tilde{F}}_{c}(t)={\tilde{F}}_{c}(0)-\frac{v_{a}p_{a}^{s}+v_{r}p_{r}^{s}}{a}t=1-\frac{v_{a}p_{a}^{s}+v_{r}p_{r}^{s}}{a}t\equiv 1-S_{c}t,$$

where $-S_{c}$ is the slope of the decrease in fluorescence in the central window.

At these early times, the flanking windows capture all the neurofilaments that exit the central window because these filaments do not have sufficient time to pass through the flanking windows and exit them on the other side. In this case, the quantities of fluorescent neurofilament polymer that leave the central window anterogradely and retrogradely per second (i.e., the fluxes $j_{a}$ and $j_{r}$) are given by the increase in the neurofilament content $F_{d}$ and $F_{p}$ in the flanking windows. Normalized to the initial content of fluorescent neurofilament polymer in the central window [i.e., ${\tilde{F}}_{p}(t)\equiv F_{p}(t)/F_{c}(0)$ and ${\tilde{F}}_{d}(t)\equiv F_{d}(t)/F_{c}(0)$], the rates of increase in the flanking windows become the following:

$$\frac{d{\tilde{F}}_{p}}{dt}=-\frac{j_{r}\left(x_{p},t\right)}{\rho a},$$

(7) $$\frac{d{\tilde{F}}_{d}}{dt}=\frac{j_{a}(x_{d},t)}{\rho a}.$$

As shown above for the central window at early times after activation, Taylor expansion also reveals a linear dependence of the normalized fluorescent neurofilament polymer content in the flanking windows, as follows:

$${\tilde{F}}_{p}(t)={\tilde{F}}_{p}(0)-\frac{j_{r}^{s}}{\rho a}t=\frac{p_{r}v_{r}}{a}t\equiv S_{p}t,$$

(8) $${\tilde{F}}_{d}(t)={\tilde{F}}_{d}(0)+\frac{j_{a}^{s}}{\rho a}t=\frac{p_{a}v_{a}}{a}t\equiv S_{d}t,$$

where $S_{p}$ and $S_{d}$ are the slopes of the linear increase in fluorescence in the proximal and distal flanking windows, respectively (Fig. 1*C*).

Combining Equation 3 and Equation 8, we can express the average velocity in terms of the length of the central window and the slopes of the linear increase in the fluorescence in the flanking windows, as follows:

(9) $$\bar{v}=a(S_{d}-S_{p}),$$

and the ratio of the number of anterograde and retrograde moving neurofilaments in terms of the ratio of these slopes, as follows:

(10) $$\frac{j_{a}^{s}}{j_{r}^{s}}=\frac{S_{d}}{S_{p}}.$$

Importantly, these expressions only apply at early times after activation during which fluorescent neurofilaments enter the flanking window at a constant rate and do not leave. The duration of this period of linearity depends on the lengths of the central and flanking windows, increasing with increasing window length. We demonstrated this dependence for the central window in the pulse-escape paradigm previously (Li et al., 2014). The dependence arises because with increasing central window length, there is an increasing probability that on-track filaments will pause before leaving the window, resulting in a slower rate of departure of the on-track filaments. The decay departs from linearity as the on-track fluorescent filaments in the central window become depleted. For the flanking windows, the duration of the period of linearity depends on the lengths of the flanking windows but is also limited by the duration of the linear decay from the central window for the reasons given above. The dependence on the length of the flanking windows arises because, for increasing window lengths, it takes longer for the fluorescent filaments to move through and begin to depart from the other end. Once filaments start to depart the flanking windows, the rate of increase of fluorescence in the flanking windows will decline making the time course of the fluorescence nonlinear. We can test experimentally whether such a limitation has occurred by recording the fluorescence for flanking windows of increasing size. If increasing the window size does not result in an increase in the fluorescence content, then we know that all labeled neurofilaments that entered the flanking window are still residing in that window. Practically speaking, the flanking window size is limited in our experiments by the camera field of view. However, where necessary, we can predict the measured fluorescence for flanking windows of infinite window size with an extrapolation function extracted from our model of neurofilament transport, as described below.

Note in Equation 9 that the velocity is given by the absolute difference between the slopes in the flanking windows. Thus, if the flanking windows are not long enough to capture all the fluorescent filaments departing from the central window during the chosen time window, then this will lead to an underestimate of velocity. In contrast, the expression for the directionality in Equation 10 is robust to flanking window size because it is given by the ratio of the slopes in the flanking windows, not the absolute difference between them. Thus, estimates of directionality, which are the focus of this article, are relatively insensitive to flanking window size.

### Modeling

Pulse-spread experiments were simulated computationally using a stochastic implementation of the six-state kinetic model of neurofilament transport described previously (Li et al., 2014; Walker et al., 2019). Fluorescent neurofilaments that depart the activated region contribute transiently to the fluorescence in the flanking windows as they pass through. As we increase the window lengths, the fraction of fluorescent neurofilaments that are captured in a certain period increases and then plateaus at a window size that is sufficient to capture all the neurofilaments that departed the activated region during that period. We do not have an analytical solution for this relationship, but empirically we find that it can be described by the following function:

(11) $${\tilde{F}}_{p,d}^{\text{actual}}(b)={\tilde{F}}_{p,d}^{\text{total}}\left(1-\text{exp}(-\beta_{p,d}b)\right),$$

where ${\tilde{F}}_{p,d}^{\text{actual}}(b)$ is the actual fluorescence in the proximal and distal flanking windows at time *t*, b is the window length, and ${\tilde{F}}_{p,d}^{\text{total}}$ is the total fluorescence that entered these windows (i.e., the fluorescence that would be captured by flanking windows of infinite length).

For our simulations, we used the first 4 min after postactivation dark-state relaxation, which is the same period that we used to determine the slopes $S_{p}$ and $S_{d}$ described above. Using the curve fit function of the SciPy/optimize package (Virtanen et al., 2020), we determined the parameters ${\tilde{F}}_{p,d}^{\text{total}}$ and $\beta_{p,d}$, which provided the best fit of Equation 11 to the experimental data ${\tilde{F}}_{p,d}^{\text{actual}}(b)$. We constrained the fitting function to pass through zero for zero-length windows and to reach a fixed rate at large window size (i.e., when the true fluorescent content ${\tilde{F}}_{p,d}^{total}$ was reached). We then estimated the linear slopes $S_{p}$ and $S_{d}$ from these curve fits by dividing the fluorescence at plateau ${\tilde{F}}_{p,d}^{\text{total}}$ by the time.

### Experimental design and statistical analysis

All observations were repeated multiple times to ensure reproducibility and then were subjected to statistical analysis. For reasons of colony management, we used exclusively male mice for our pulse-spread analysis at different ages and for our experiments using metabolic inhibitors. A separate comparison of male and female mice at 8 weeks of age revealed no statistically significant differences in the pulse-spread kinetics. Each experimental group consisted of at least 70 individual axons taken from six to seven different regions of at least five nerves, and each nerve came from a different animal. The only exception to this was for the nerves treated with metabolic inhibitors, when only one region of each nerve was imaged because of concerns about the effect of the inhibitors on nerve viability. For all measures (flux, velocity, and directionality), the axon-to-axon variability (SEM) within each nerve was equal to or greater than the nerve-to-nerve (i.e., animal-to-animal) variability. Thus, each axon was considered an independent replicate. All data were presented as box-and-whisker plots showing minimum, maximum, median, first and third quartiles, along with all individual data points. Normality was determined for each group using the Shapiro–Wilk test. Since many comparisons were made among all groups, data for each measure were pooled into Kruskal–Wallis nonparametric ANOVAs with *post hoc* Mann–Whitney pairwise comparisons corrected for type I error using the Benjamini and Hochberg (1995) method. The three Kruskal–Wallis tests encompassed the neurofilament transport rates, the neurofilament population velocities, and axon diameter. Correlations between axon diameter and neurofilament transport kinetics were assessed with the Pearson’s correlation test. Anterograde bias was tested using a Wilcoxon signed-rank test against a hypothetical median value of 0.5 (50% anterograde, 50% retrograde). The α was set to 0.05 for all comparisons. Benjamini–Hochberg-corrected *p*-values are included alongside any comparison between populations.

## Results

### The pulse-spread experimental paradigm

We previously developed a population-based fluorescence photoactivation strategy called the “pulse-escape” technique to analyze the long-term pausing behavior of neurofilaments (Trivedi et al., 2007). In that method, neurofilaments containing a photoactivatable neurofilament protein are marked by photoactivation of a short segment of axon, and then the loss of fluorescence from the activated window is analyzed over time. We have shown that this method can yield kinetic predictions of the long-term and short-term pausing behaviors (Li et al., 2014; Monsma et al., 2014; Walker et al., 2019). However, a significant limitation of this method is that it is blind to the directionality of neurofilament movement. In other words, tracking the loss of fluorescence in the activated window does not reveal the direction in which those neurofilaments departed. To address this, we present a variation on this approach, which we term “pulse-spread.” For this method, we define adjacent proximal and distal windows that flank the activated region and quantify the increase in fluorescence in the flanking windows at short times after photoactivation (Fig. 1*B*).

Computational simulation of a pulse-spread experiment predicts that the fluorescence should increase initially as activated neurofilaments are transported into the flanking windows from the central activation window. After some minutes, the rate of increase in the fluorescence is predicted to slow and then plateau as the entrance of neurofilaments from the central window is offset by the exit of filaments on the other side. Then at later times the fluorescence is predicted to decline as fluorescent filaments become depleted from the central window, and those in the flanking regions continue to exit and disperse along the length of the axon. For the present analysis, we focused exclusively on the rate of increase at short times (Fig. 1*C*).

To analyze neurofilament transport in myelinated axons, tibial nerves were dissected from 8-week-old *hThy1-paGFP-NFM* mice, which express neurofilament protein M tagged with paGFP-NFM in neurons under control of the human Thy1 promoter (Walker et al., 2019). The expression level of the paGFP-NFM fusion protein in these mice is 1.6% of the endogenous NFM in the sciatic nerve (Walker et al., 2019). The nerves were desheathed and mounted on a glass coverslip in a heated closed-bath imaging and perfusion chamber on the stage of an inverted microscope. The preparation was perfused with oxygenated saline and observed using spinning disk confocal microscopy. By tracking mitochondrial motility, we confirmed that the nerves remain healthy under these conditions for at least 3 h (Walker et al., 2019). Myelinated axons can be identified by the presence of a myelin sheath, which is visible under bright-field illumination (Fig. 1*D*).

To perform a pulse-spread experiment, we activated the paGFP fluorescence in a 40-μm-long window of nerve using violet light and acquired time-lapse images of the photoactivated axons at 30 s intervals. The long length of the activation window was necessary to maximize the length of time over which the fluorescence in the flanking windows increased linearly (Boyer et al., 2020; see Materials and Methods). Single neurofilaments cannot be resolved because they are packed densely, and their individual fluorescence is very low. However, since each axon contains thousands of neurofilaments, the summation of the weak fluorescence of the individual filaments can be detected. After activation of the central window, the sharp proximal and distal edges of the activated region blur slowly as fluorescent neurofilaments are transported out, resulting in a slow spreading of the activated fluorescence (Fig. 1*E*, 0–30 min, Movie 1). The slow rate of spreading reflects the kinetic behavior of the filaments, which spend most of their time pausing and move only infrequently (Trivedi et al., 2007). Importantly, this spreading occurs at both the proximal and distal ends of the activated region, indicating that the movement in these axons is bidirectional.

Movie 1. Pulse-spread experiment in 8-week-old male tibial nerve. Time zero corresponds to 1 min postactivation, after relaxation of the paGFP from the dark state (see Materials and Methods). The length of each activated axonal segment was 40 μm. The time-stamp format is minutes:seconds. There is noticeable photobleaching during the 30 min movie; our analyses were performed during the first 4 min, and our measurements of fluorescence intensity were corrected for photobleaching. Proximal is left; distal is right. 10.1523/ENEURO.0138-22.2022.video.1

**Figure 2.**
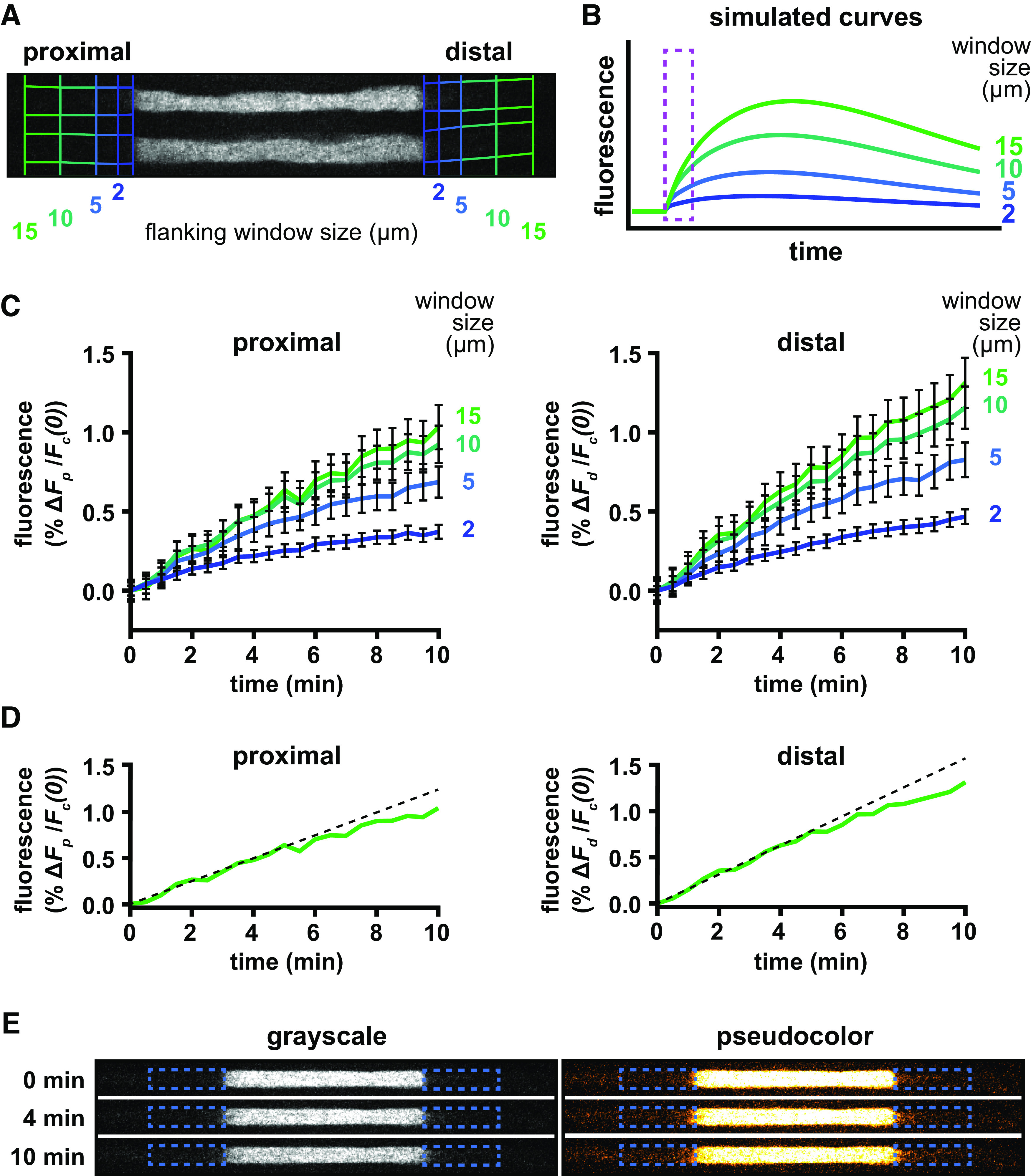
Determination of flanking window size. ***A***, Example of a single axon imaged immediately after photoactivation, showing the four different lengths of flanking windows (2, 5, 10, and 15 μm) used in our analysis. ***B***, Graph of the fluorescence in distal flanking windows of varying size generated by computational simulation (see Materials and Methods), showing the predicted rise and fall of the fluorescence. For our analyses, we measured the increase in the fluorescence at short times (marked by the dashed magenta box). ***C***, Fluorescence increases measured in proximal and distal flanking windows of varying sizes from pulse-spread experiments conducted in the tibial nerves of 8-week-old male mice (*n* = 7 nerves, *n* = 92 axons). ***D***, Approximation of the rate of fluorescence increase by linear regression (dashed black line) of the first 4 min of data using 15 μm flanking windows (solid green line, reproduced from ***C***). ***E***, An example of one axon at 0, 4, and 10 min after activation shown in grayscale (left) and pseudocolor (right). Proximal is left, and distal is right. We measured the rate of increase of the fluorescence in the proximal and distal flanking regions (dashed blue boxes) during the first 4 min after activation. This was a very small but measurable fraction of the activated fluorescence.

To quantify the spread of the fluorescent filaments from the activated region in each axon, we measured the change in fluorescence intensity in proximal and distal flanking windows in the time-lapse movies. To explore the effect of flanking window size, we measured the fluorescence in flanking windows of increasing length on both the proximal (toward the soma) and distal (toward the axon tip) sides of the central activation window (Fig. 2*A*). Computational simulations of pulse-spread experiments predict that lengthening the flanking windows will increase the total amount of fluorescence captured and extend the initial period over which the fluorescence increase remains linear (Fig. 2*B*; see Materials and Methods). Figure 2*C* plots the change in fluorescence in the proximal and distal windows (Δ*F_p_* and Δ*F_d_*, respectively) normalized to the initial fluorescence of the central activation window [*F_c_*(0)] for window lengths of 2, 5, 10, and 15 μm. With a 15 μm window, the curves were linear out to at least 4 min (Fig. 2*D*). We therefore chose to use a flanking window size of 15 μm and to measure the slope in the first 4 min for all subsequent pulse-spread experiments.

### Neurofilament transport is bidirectional with an anterograde bias

**Figure 3.**
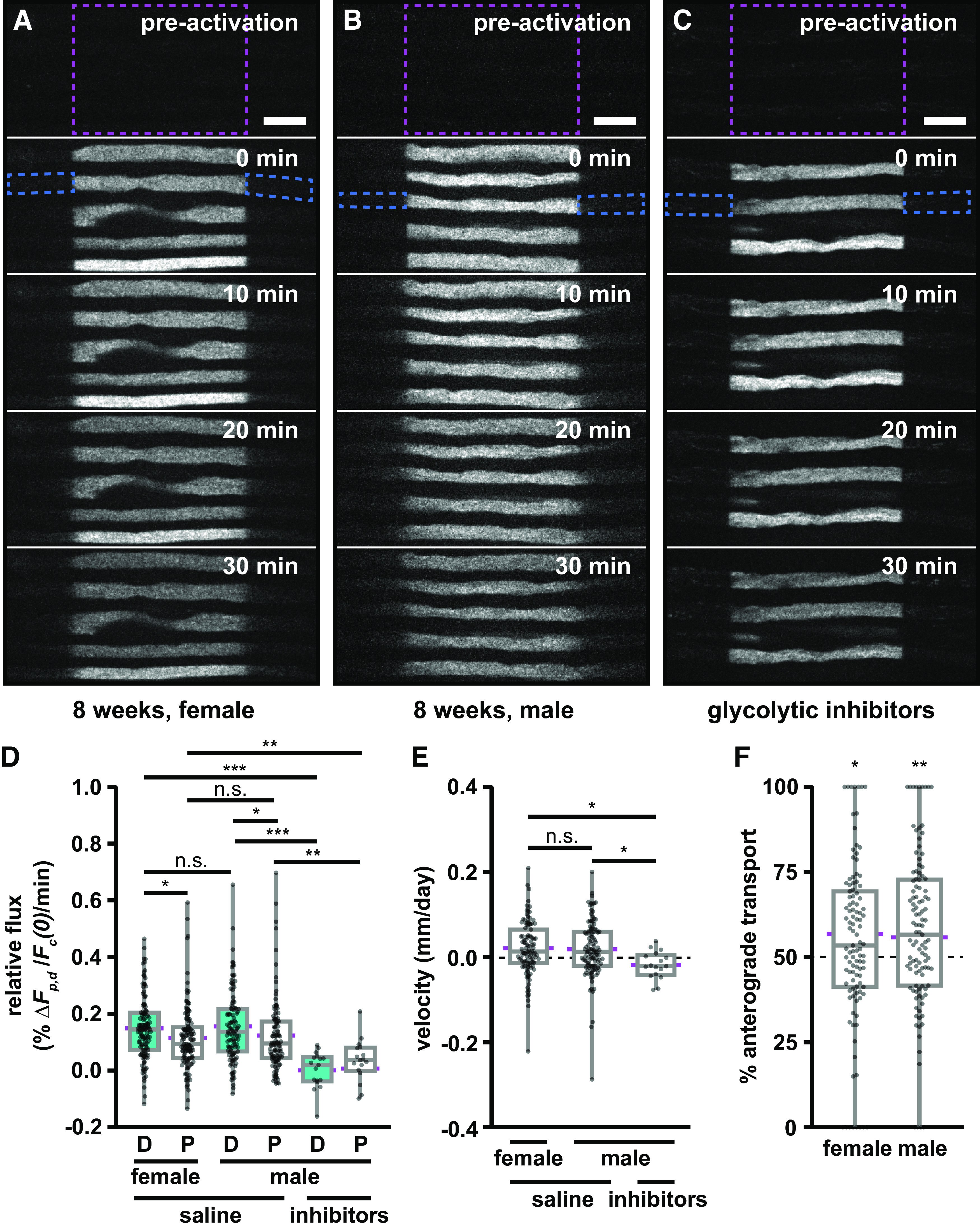
Neurofilament transport is bidirectional with an anterograde bias. ***A***, ***B***, Frames from representative time-lapse image series of pulse-spread experiments in the tibial nerve of an 8-week-old female (***A***) and an 8-week male (***B***) *hThy1-paGFP-NFM* mouse. ***C***, A representative time-lapse image of a pulse-spread experiment in a nerve from an 8-week-old male *hThy1-paGFP-NFM* mouse perfused with saline containing the glycolytic inhibitors 2-deoxy-d-glucose and sodium iodoacetate. The activated regions (40 μm in length) are marked by dashed magenta boxes: proximal is left, distal is right throughout. The dashed blue boxes mark the proximal and distal flanking regions for one axon at *t* = 0 min. Note that there is some loss of fluorescence because of photobleaching in these raw images. Our quantitative analysis was confined to the first 4 min after activation, and the data were corrected for photobleaching (see Materials and Methods). Scale bar, 10 μm. ***D***, Quantification of relative flux of the fluorescence in the proximal (P) and distal (D) flanking regions (each 15 μm in length) in the first 4 min following photoactivation (*n* = 107 axons in six nerves for the female group; *n* = 111 axons in eight nerves for the male group; *n* = 17 axons in five nerves for the male group treated with inhibitors). ***E***, Neurofilament population transport velocity (positive is anterograde, negative is retrograde). ***F***, Percentage of neurofilament transport in the anterograde direction, calculated as the ratio of the distal relative flux to the sum of the distal and proximal relative flux. Significance in ***F*** is tested against a theoretical population with a mean of 50% and an equivalent SD. Each data point in ***D***, ***E***, and ***F*** represents the measurement for a different axon. The boxes show the median, upper, and lower quartiles, and the whiskers show the minimum and maximum. The magenta points on either side of the boxes show the sample mean. The mean velocity and mean percentage anterograde were calculated using the average relative proximal and distal fluxes. n.s. *p* > 0.05, * *p* ≤ 0.05, ** *p* ≤ 0.01, *** *p* ≤ 0.001.

To quantify the directional bias, we performed pulse-spread experiments in the tibial nerves of 8-week-old mice and measured the rate of increase in the fluorescence in the proximal and distal flanking windows, *S_p_* and SD (Fig. 3). These experiments proved technically challenging because of the low expression level of the paGFP-NFM in these axons and the slow rate of neurofilament transport, which resulted in very low fluorescence intensities in the flanking regions. For example, the average fluorescence in the distal 15 μm flanking window at 4 min after photoactivation was 1% of the fluorescence in the central window immediately after activation (Fig. 2*C*). To address this, we used 4 s exposures and analyzed 84–135 axons from 5 to 11 animals for each experimental condition.

To control for sex-specific effects, we compared separate cohorts of male and female mice (Fig. 3*A*,*B*, Movies 1, 2, respectively). A significant fraction of the filaments moved retrogradely in both cohorts, but in each case the distal (anterograde) rate was higher than the proximal (retrograde) rate (female, *p* = 0.0102; male, *p* = 0.0182; Fig. 3*D*). Taking the ratio of these rates, we can obtain the directional bias (Eq. 10). The mean proportion moving anterogradely was 56% anterograde in the males and 57% in the females (Fig. 3*F*). These proportions were not significantly different from each other (*p* = 0.386), but both were significantly different from a theoretical value of 50% (female *p* = 0.044; male *p* = 0.00199). Thus, while there is considerable variation from axon to axon on the short timescale of these measurements (see Discussion), the data confirm that neurofilament transport is biased in the anterograde direction. Notably, however, ∼43–44% of the filaments moved retrogradely. Since we did not establish a difference between male and female mice, we performed all subsequent experiments on male mice only.

Movie 2. Pulse-spread experiment in 8-week-old female tibial nerve. Time zero corresponds to 1 min postactivation, after relaxation of the paGFP from the dark state (see Materials and Methods). The length of each activated axonal segment was 40 μm. The time stamp format is minutes:seconds. There is noticeable photobleaching during the 30 min movie; our analyses were performed during the first 4 min, and our measurements of fluorescence intensity were corrected for photobleaching. Proximal is left; distal is right. 10.1523/ENEURO.0138-22.2022.video.2

To confirm that the spread of the fluorescence was because of active transport, we performed pulse-spread experiments in nerves that had been treated with saline containing 5.6% 2-deoxy-d-glucose and 0.5 mm sodium iodoacetate, which are inhibitors of glycolysis. We have shown previously that this treatment effectively inhibits neurofilament transport in cultured neurons and tibial nerves (Koehnle and Brown, 1999; Trivedi et al., 2007; Monsma et al., 2014; Walker et al., 2019). This is consistent with reports for other cargoes that glycolysis is the primary source of ATP to support axonal transport (Ochs and Smith, 1971; Zala et al., 2013; Hinckelmann et al., 2016). As expected, the glycolytic inhibitors blocked the bidirectional spread of the neurofilament fluorescence, leaving the proximal and distal ends of the activated regions sharply delineated even after 30 min (Fig. 3*C*, Movie 3). Quantitatively, we observed a 98% and 71% reduction in the anterograde and retrograde rates, respectively, which was statistically significant (*p* = 0.0000050 and *p* = 0.0049, respectively; Fig. 3*D*). Thus, the spreading of the fluorescence is an active transport process.

Movie 3. Pulse-spread experiment in 8-week-old male tibial nerve with glycolytic inhibition. Time zero corresponds to 1 min postactivation, after relaxation of the paGFP from the dark state (see Materials and Methods). The length of each activated axonal segment was 40 μm. The time-stamp format is minutes:seconds. There is noticeable photobleaching during the 30 min movie; our analyses were performed during the first 4 min, and our measurements of fluorescence intensity were corrected for photobleaching. Note that the proximal and distal ends of the activated regions remain sharp, with no spread of fluorescence into the flanking regions. Proximal is left; distal is right. 10.1523/ENEURO.0138-22.2022.video.3

As shown in the Modeling subsection of Materials and Methods, the initial linear slopes in the proximal and distal flanking windows can also yield an average velocity for the neurofilament population, with a positive velocity representing net anterograde transport and a negative velocity representing net retrograde transport (see Materials and Methods). We found that the male and female mice had average neurofilament population velocities in the tibial nerve of 0.018 and 0.021 mm/d, respectively, which were not significantly different (Fig. 3*E*; *p* = 1). Radioisotopic pulse-labeling studies have yielded estimates of 0.6 mm/d in the proximal sciatic nerve, slowing approximately fivefold to 0.12 mm/d in the distal sciatic nerve. There are no published pulse-labeling studies that have extended into the tibial nerve, but our measurements here using the pulse-spread method suggest that this slowing of the neurofilament transport rate along the sciatic nerve may continue into its distal branches (see Discussion).

### Neurofilament transport slows during postnatal development

The expansion of axon caliber during postnatal development is driven by an accumulation of axonal neurofilaments, triggered by outside-in signaling from the myelinating cells (Hsieh et al., 1994; Sánchez et al., 2000; Garcia et al., 2003; Monsma et al., 2014). It has been proposed that this accumulation is caused by an increase in neurofilament gene expression and a spatial and temporal slowing of neurofilament transport (Hoffman et al., 1983, 1984, 1985, 1987; Muma et al., 1991; Hoffman, 1995). Computational modeling of the slowing of neurofilament transport in mouse sciatic nerve suggested that the slowing could be caused by an increase in time spent pausing or a shift in the balance of anterograde and retrograde movement (Jung and Brown, 2009).

**Figure 4.**
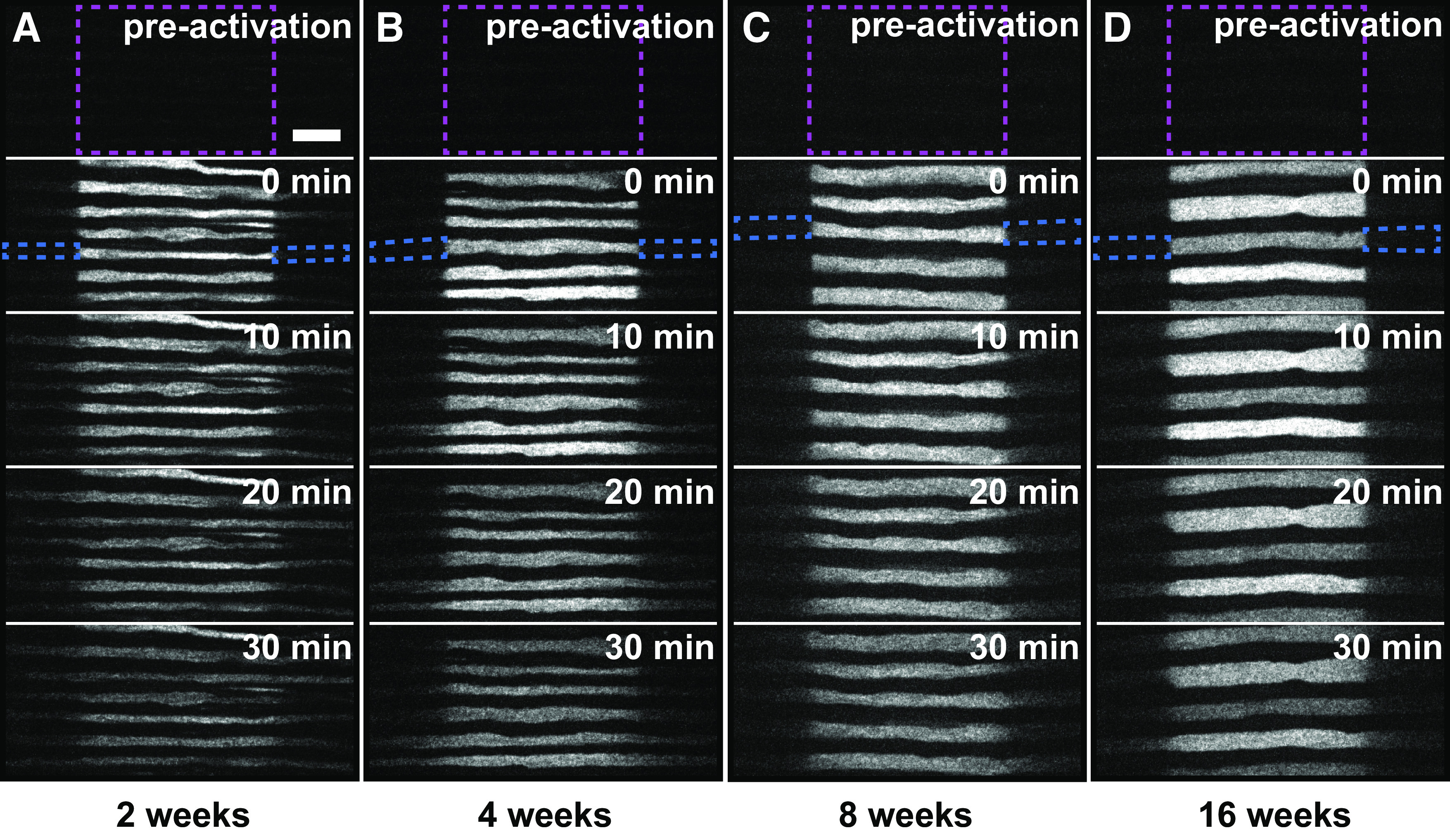
Pulse-spread time-lapse image series of axons at different ages. ***A–D***, Frames from representative time-lapse image series of pulse-spread experiments in the tibial nerves of male *hThy1-paGFP-NFM* mice that were 2 (***A***), 4 (***B***), 8 (***C***), and 16 weeks (***D***). The data for 8 weeks is reproduced from Figure 3 to allow side-by-side comparison with the other ages. The top frame represents the preactivation image. The dashed magenta box marks the activated region. The dashed blue boxes mark the proximal and distal flanking regions for one axon at *t* = 0 min. Note that there is some loss of fluorescence because of photobleaching in these raw images. Our quantitative analysis was confined to the first 4 min after activation, and the data were corrected for photobleaching (see Materials and Methods). Proximal is left; distal is right. Scale bar, 10μm.

**Figure 5.**
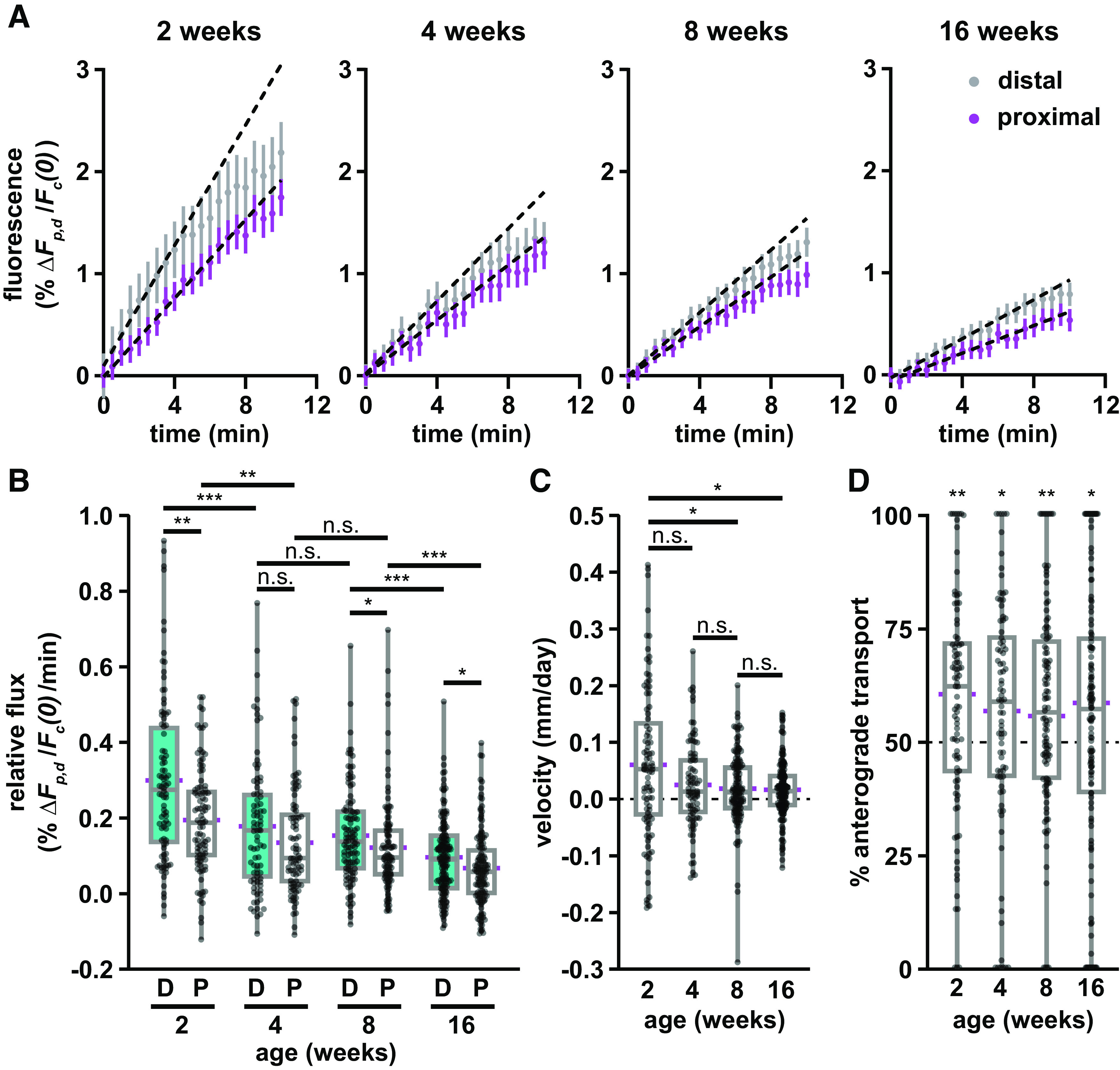
Neurofilament transport slows with age. ***A***, Fluorescence measurements from the proximal (magenta) and distal (gray) flanking windows of axons from each age group, showing mean (dot) and SEM (bars) of the full dataset (92 axons in seven nerves at 2 weeks; 84 axons in six nerves at 4 weeks; 111 axons in eight nerves at 8 weeks; 135 axons in seven nerves at 16 weeks), with respect to time after activation. Dashed black lines show linear regressions fit to the first 4 min of each dataset. ***B***, Quantification of the relative flux of the fluorescence in the proximal (P) and distal (D) flanking regions in the first 4 min following photoactivation in nerves from male *hThy1-paGFP-NFM* mice 2, 4, 8, and 16 weeks of age. The 8 week data are the same data shown in Figure 3. ***C***, Neurofilament population transport velocity variation with age (positive is anterograde; negative is retrograde). ***D***, Percentage of neurofilament transport in the anterograde direction for each age group, calculated as the ratio of the distal relative flux to the sum of the distal and proximal relative flux. Significance in ***D*** is tested against a theoretical population with a mean of 50% and an equivalent SD. Each data point in ***B***, ***C***, and ***D*** represents the measurement for a different axon. The boxes show the median, upper, and lower quartiles, and the whiskers show the minimum and maximum. The magenta points on either side of the boxes show the sample mean. The mean velocity and the mean percentage anterograde were calculated using the average relative proximal and distal fluxes. n.s. *p* > 0.05, * *p* ≤ 0.05, ** *p* ≤ 0.01, *** *p* ≤ 0.001.

To investigate the slowing mechanism, we performed pulse-spread experiments on tibial nerves of mice 2, 4, and 16 weeks of age, adding to the data described above at 8 weeks. As expected, the axons were relatively slender at 2 weeks and increased in caliber with age (Fig. 4). Bidirectional spreading of the fluorescence from the activated region was evident at all ages but was substantially greater in the youngest nerves and declined with age (Fig. 4, Movies 4, 5, 1, 6). Figure 5*A* shows the average fluorescence change in the proximal and distal flanking windows over time with linear regression lines to show the slopes in the first 4 min, and Figure 5*B* shows the column scatter plots in which each data point is the slope calculated in the proximal or distal window for one axon. There was a progressive decrease in both anterograde and retrograde transport with age. At all ages, the average relative transport flux in the distal (anterograde) direction was greater than in the proximal (retrograde) direction, and this was statistically significant at 2, 8, and 16 weeks of age (*p* = 0.0052, *p* = 0.0182, and *p* = 0.0374, respectively). Between 2 and 4 weeks of age, the fluxes decreased by 40% (*p* = 0.000363) in the anterograde direction and by 31% (*p* = 0.00,461) in the retrograde direction. The fluxes decreased further from 4 to 8 weeks, but these differences were not statistically significant (anterograde, *p* = 0.698; retrograde, *p* = 0.697). Between 8 and 16 weeks, anterograde transport decreased by 38% (*p* = 0.000873), and retrograde by 44% (*p* = 0.000945). Overall, anterograde and retrograde neurofilament transport decreased by 68% and 65%, respectively, from 2 to 16 weeks of age. Considering the age-dependent decrease in both anterograde and retrograde neurofilament transport, we investigated whether there was a corresponding decrease in overall neurofilament population velocity (Fig. 5*C*). The average velocity had a net anterograde bias at all ages but declined progressively by 73% from 2 to 16 weeks. While the decline was not statistically significant from 2 to 4 weeks (*p* = 0.105), 4 to 8 weeks (*p* = 0.835), or 8 to 16 weeks (*p* = 0.915), it was significant from 2 to 8 weeks (*p* = 0.0477) and 2 to 16 weeks (*p* = 0.0179). The anterograde bias was statistically significant at all ages (*p* = 0.0029 at 2 weeks; *p* = 0.030 at 4 weeks; *p* = 0.0020 at 8 weeks; *p* = 0.040 at 16 weeks; Fig. 5*D*). However, the magnitude of this bias was fairly stable, fluctuating between 56% and 61%, with no statistically significant difference between ages.

Movie 4. Pulse-spread experiment in 2-week-old male tibial nerve. Time zero corresponds to 1 min postactivation, after relaxation of the paGFP from the dark state (see Materials and Methods). The length of each activated axonal segment was 40 μm. The time-stamp format is minutes:seconds. There is noticeable photobleaching during the 30 min movie; our analyses were performed during the first 4 min, and our measurements of fluorescence intensity were corrected for photobleaching. Proximal is left; distal is right. 10.1523/ENEURO.0138-22.2022.video.4

Movie 5. Pulse-spread experiment in 4-week-old male tibial nerve. Time zero corresponds to 1 min postactivation, after relaxation of the paGFP from the dark state (see Materials and Methods). The length of each activated axonal segment was 40 μm. The time-stamp format is minutes:seconds. There is noticeable photobleaching during the 30 min movie; our analyses were performed during the first 4 min, and our measurements of fluorescence intensity were corrected for photobleaching. Proximal is left; distal is right. 10.1523/ENEURO.0138-22.2022.video.5

Movie 6. Pulse-spread experiment in 16-week-old male tibial nerve. Time zero corresponds to 1 min postactivation, after relaxation of the paGFP from the dark state (see Materials and Methods). The length of each activated axonal segment was 40 μm. The time-stamp format is minutes:seconds. There is noticeable photobleaching during the 30 min movie; our analyses were performed during the first 4 min, and our measurements of fluorescence intensity were corrected for photobleaching. Proximal is left; distal is right. 10.1523/ENEURO.0138-22.2022.video.6

**Figure 6.**
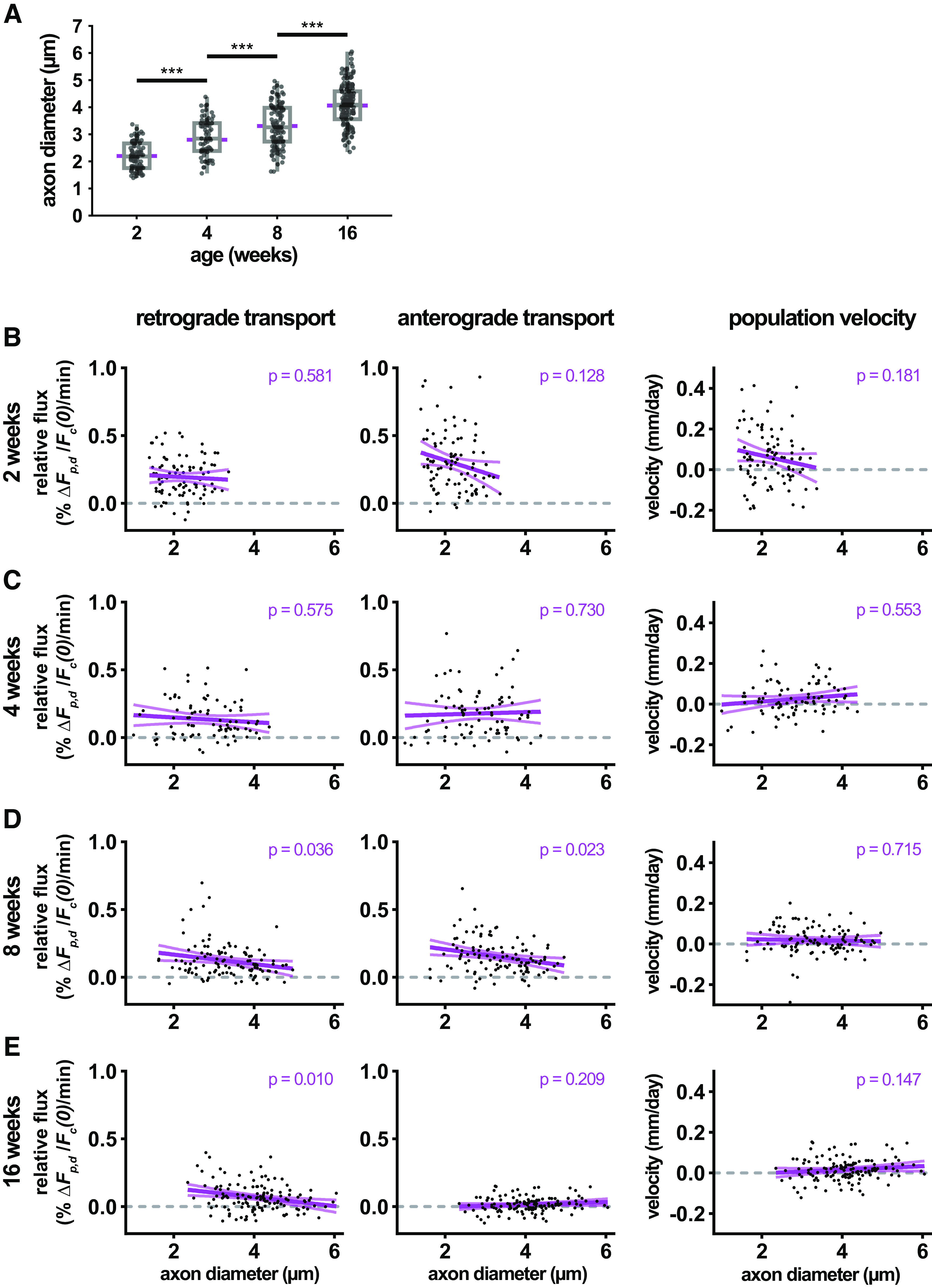
Neurofilament transport velocity does not correlate with axon diameter. ***A***, Quantification of axon diameter in photoactivated axons at 2, 4, 8, and 16 weeks of age. ***B–E***, Scatterplots of retrograde and anterograde neurofilament transport flux and neurofilament population velocity versus axon diameter at 2, 4, 8, and 16 weeks of age. Linear regression of scatterplots with 95% confidence intervals are shown in magenta, along with the *p*-values of each regression. The data in this figure come from the axons analyzed in Figure 5.

A unique feature of the pulse-spread analysis is that it permits the analysis of neurofilament transport in single axons, and thus it is possible to ask for the first time whether there is any correlation between transport velocity and axon caliber. The average myelinated axon diameter increased approximately linearly from 2.2 μm at 2 weeks to 4.1 μm at 16 weeks, with individual axons across these ages ranging from 0.995 to 6.046 μm in diameter (Fig. 6*A*). While there was considerable scatter in the data, the slope of the regression line was negative for retrograde rates at all ages, and for anterograde rates at 2 and 8 weeks (Fig. 6*B–E*). These slopes were not statistically significant at 2 and 4 weeks, but they were statistically significant for the anterograde and retrograde slopes at 8 weeks (*p* = 0.036 and 0.023, respectively), and for the retrograde slopes at 16 weeks (*p* = 0.010). Thus, while there is a trend toward decreasing transport rates with increasing axon diameter, this was not consistent across all ages. Moreover, there was no significant correlation between the neurofilament population transport velocity and axon caliber at any age.

### Proximal-to-distal slowing of neurofilament transport

**Figure 7.**
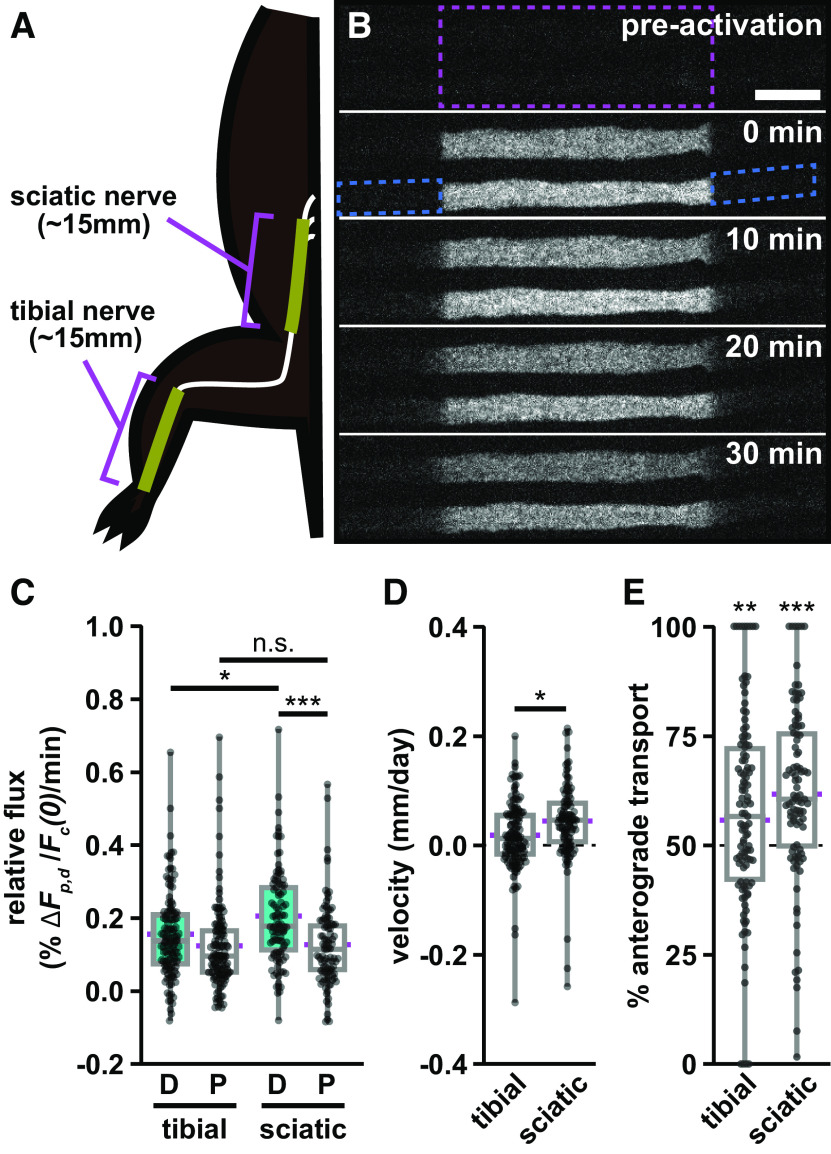
Anterograde neurofilament flux is higher in proximal nerve segments. ***A***, Diagram showing the relative location of the sciatic and tibial nerve segments in relation to the spine and hindlimb. ***B***, Frames from a representative time-lapse image series of a pulse-spread experiment in the proximal sciatic nerve. The activated region is marked by the dashed magenta box, and the proximal and distal flanking regions are marked by the dashed blue boxes for one axon at *t* = 0 min. Note that there is some loss of fluorescence because of photobleaching in these raw images. Our quantitative analysis was confined to the first 4 min after activation, and the data were corrected for photobleaching (see Materials and Methods). Proximal is left, and distal is right. Scale bar, 10 μm. ***C***, Quantification of the relative fluorescence flux in the distal (D) and proximal (P) flanking windows in the first 4 min following photoactivation (111 axons in 8 nerves for the tibial; 95 axons in 10 nerves for the sciatic). The tibial nerve data used for comparison is the 8-week-old male cohort shown in Figures 3 and 5. ***D***, Neurofilament population transport velocity in the sciatic and tibial nerves (positive is anterograde; negative is retrograde). ***E***, Percentage of neurofilament transport in the anterograde direction in the tibial and sciatic nerves. Significance in ***E*** is tested against a theoretical population with a mean of 50% and an equivalent SD. The boxes show the median, upper, and lower quartiles, and the whiskers show the minimum and maximum. The magenta points on either side of the boxes show the sample mean. The mean velocity and mean percentage anterograde were calculated using the average relative proximal and distal fluxes. n.s. *p* > 0.05, * *p* ≤ 0.05, ** *p* ≤ 0.01, *** *p* ≤ 0.001.

Radioisotopic pulse-labeling studies have reported both a spatial and temporal slowing of neurofilament transport in rodent sciatic nerve (Hoffman et al., 1985; Xu and Tung, 2001). Teasing apart the relative contribution of these two effects is complicated because the animals age and grow during the pulse-labeling experiments, which last weeks or months. In contrast, the pulse-spread method measures the transport velocity locally on a timescale of minutes, allowing us to isolate temporal and spatial influences. To test whether this method can detect a spatial slowing along the nerve, we compared our measurements in the tibial nerve with axons in the sciatic nerve, which is more proximal to the spinal cord (Fig. 7*A*,*B*, Movie 7). For this experiment, we used mice that were 8 weeks old. The average flux in the distal (anterograde) direction was greater than in the proximal (retrograde) direction, and this was statistically significant in both the tibial and sciatic nerves (*p* = 0.0182, *p* = 0.0000799, respectively). The relative anterograde flux was 31% higher in the sciatic than in the tibial (Fig. 7*C*; *p* = 0.0103), but the retrograde transport flux was not significantly different (*p* = 0.568). Consistent with this difference in anterograde flux, the neurofilament population velocity was ∼0.044 mm/d in the sciatic nerve, 2.4-fold higher than the tibial nerve at the same age (Fig. 7*D*; *p* = 0.0130). The anterograde bias was statistically significant in both the sciatic and tibial nerves (Fig. 7*E*; *p* = 0.000000383 and *p* = 0.00199, respectively). The magnitude of the bias was 62% in the sciatic nerve and 56% in the tibial nerve, though this difference was not statistically significant. In conclusion, our results confirm that neurofilament transport slows in a proximal-to-distal manner and suggest that this may be due specifically to a decrease in anterograde neurofilament transport.

Movie 7. Pulse-spread experiment in 8-week-old male sciatic nerve. Time zero corresponds to 1 min postactivation, after relaxation of the paGFP from the dark state (see Materials and Methods). The length of each activated axonal segment was 40 μm. The time-stamp format is minutes:seconds. There is noticeable photobleaching during the 30 min movie; our analyses were performed during the first 4 min, and our measurements of fluorescence intensity were corrected for photobleaching. Proximal is left; distal is right. 10.1523/ENEURO.0138-22.2022.video.7

### Modeling estimation of neurofilament transport rates

**Figure 8.**
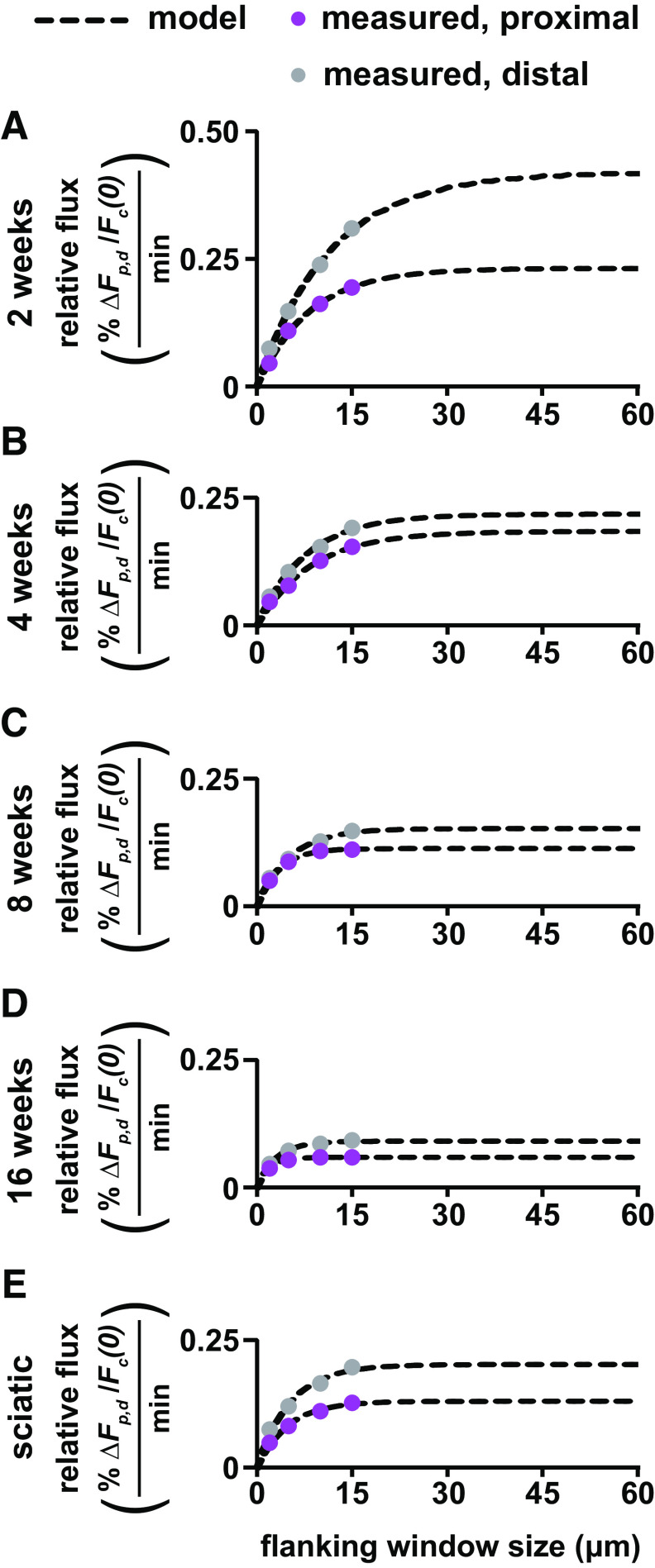
Extrapolation of relative flux to infinite flanking window size using mathematical modeling. ***A–E***, Population averages of the relative fluorescence flux in the proximal and distal flanking regions measured over the first 4 min plotted against flanking window size for 2–16-week-old tibial nerve and 8-week-old sciatic nerve. Data from the experiments shown in Figures 3, 5, and 7. Dashed black lines show the optimal fits using our mathematical model, allowing extrapolation of the relative flux to window sizes beyond the physical limit imposed by the camera field of view.

As discussed in the Materials and Methods, an assumption of the pulse-spread analysis is that we capture all the fluorescent neurofilaments that depart the activated region during the first 4 min after activation, which is the period over which we measured the rate of increase in the fluorescence $S_{d}$ and $S_{p}$ (see Materials and Methods). We used 15 μm windows, which was the largest that could be accommodated within our camera field of view. However, if some neurofilaments move out of these windows during the first 4 min, then we will underestimate the rates, which could affect the calculated directionality and transport velocity (Eqs. 9, 10). To test this assumption, we reanalyzed the axons in the pulse-spread movies of tibial and sciatic nerves (Figs. 5, 7) using flanking window sizes ranging from 2 to 15 μm (Fig. 8). In all cases, the fluorescence in the flanking windows increased as the flanking window size increased from 2 to 5 and 5 to 10 μm. At 8 and 16 weeks of age, the fluorescence in the proximal window did not increase further from 10 to 15 μm. Thus, a 10 μm window size was sufficient to capture all the retrogradely moving fluorescent neurofilaments during the first 4 min after activation in myelinated axons at 8 and 16 weeks of age. However, in the proximal and distal windows at younger ages (2 and 4 weeks) and in the distal windows at older ages (8 and 16 weeks), the fluorescence increased from 10 to 15 μm, suggesting that the 15 μm window may not have been sufficient to capture all the neurofilaments.

To estimate how much this might affect our calculations of velocity and directionality, we fitted the experimental data with our model (Eq. 11) and then extrapolated to the asymptote at larger window sizes to predict at what fluorescence intensity the curves would be expected to plateau. We then recalculated the slopes $S_{d}$ and $S_{p}$ using these extrapolated intensities and used these measurements to calculate predicted transport velocities and directionality (Table 1). At 8 and 16 weeks, the model predicts that the 15 μm windows captured essentially all the fluorescence, resulting in an underestimate of the velocity by only 22% and 11%, respectively. At 2 and 4 weeks, the model predicts that the 15 μm windows failed to capture 18–39% of the fluorescence in the proximal and distal flanking windows, resulting in a 21% overestimate of the velocity at 4 weeks and a 77% underestimate of the velocity at 2 weeks. The model also predicts that the flanking windows would need to be 30–60 μm in length to capture all the fluorescence in these younger axons (Fig. 8). As we noted in the Materials and Methods, and in contrast to the velocity, estimates of the directionality were relatively insensitive to flanking window size, differing by ≤3% at 8 and 16 weeks and by ≤6% at 2 and 4 weeks (Table 1).

**Table 1:** Comparison between modeled and actual neurofilament transport data

Group	Relative anterograde flux (% Δ*F_d_* / *F_c_*(0) /min)			Relative retrograde flux (% Δ*F_p_* / *F_c_*(0) /min)			Velocity (mm/d)			Anterograde (%)		
	Measured	Model	Difference	Measured	Model	Difference	Measured	Model	Difference	Measured	Model	Difference
Tibial nerve												
2 weeks	0.299	0.415	+39%	0.195	0.231	+18%	0.060	0.106	+77%	60.5	64.2	+6%
4 weeks	0.178	0.218	+22%	0.135	0.184	+36%	0.025	0.020	−21%	56.9	54.2	−5%
8 weeks	0.154	0.152	−1%	0.122	0.113	−7%	0.018	0.022	+22%	55.8	57.3	+3%
16 weeks	0.096	0.091	−5%	0.068	0.060	−12%	0.016	0.018	+11%	58.7	60.5	+3%
Sciatic nerve												
8 weeks	0.202	0.202	0%	0.125	0.130	+4%	0.044	0.041	−7%	61.8	60.8	−2%

Table compares the average measured neurofilament transport flux, velocity, and percentage anterograde for the data reported in Figures 5 and 7, with predicted values based on modeling an extrapolation to infinite window size. The average velocity and average percentage anterograde were calculated using the average relative proximal and distal fluxes (Eqs. 9, 10). The percentage difference (“difference”) between the experimentally derived (“measured”) and mathematically modeled (“model”) values are shown.

In conclusion, the modeling predicts an average neurofilament transport velocity in myelinated axons of the tibial nerve of 0.106 mm/d (64% anterograde) at 2 weeks, slowing about fivefold to 0.018 mm/d (61% anterograde) at 16 weeks (Table 1). The largest discrepancy between the experimental and modeled measurements was in the distal flanking window at 2 weeks. Thus, our analysis method is prone to underestimation of the velocity and directionality in young animals because of the higher neurofilament transport flux and technical limitations related to the flanking window size but is more reliable in adult animals.

## Discussion

### Neurofilament transport is bidirectional *in vivo*

The bidirectionality of neurofilament transport was first proposed in the 1990s by Griffin and colleagues (Glass and Griffin, 1991; Watson et al., 1993; Glass and Griffin, 1994) based on experiments with surgically transected nerve segments in C57BL6/Ola mice, which exhibit delayed Wallerian degeneration. Over time, neurofilaments redistributed to both ends of the cut axons, implying both anterograde and retrograde movement. These observations received little attention at the time, perhaps because they contradicted the prevailing notion that slow axonal transport was unidirectional. Our present data confirm that neurofilament transport is indeed bidirectional *in vivo* and that ∼40% of the neurofilaments move retrogradely. This falls within the range of 17–59% retrograde (average, 41%) that we have observed in seven studies on cultured neurons (Wang et al., 2000; Wang and Brown, 2001, 2010; Uchida and Brown, 2004; Alami et al., 2009; Uchida et al., 2009; Fenn et al., 2018a). Thus, bidirectional movement appears to be a fundamental property of neurofilaments, and a substantial fraction move retrogradely even in mature myelinated axons.

The conclusion that neurofilament transport is bidirectional *in vivo* is consistent with our predictions based on computational modeling of radioisotopic pulse-labeling experiments. In those experiments, a pulse of newly synthesized radioactive neurofilament proteins in neuronal cell bodies produces a wave that spreads as it propagates distally, indicating that neurofilaments move at a broad range of rates (Brown, 2014). In computational simulations, we had to assume that the movement was bidirectional to explain the extent of spreading (Brown et al., 2005; Jung and Brown, 2009). We predicted that the filaments switch repeatedly between anterograde and retrograde bouts of movement over the weeks that they spend in transit, but on average spend more time moving anterogradely, resulting in net anterograde movement. While our present data do not prove that reversals occur *in vivo*, we have observed them in cultured neurons (Fenn et al., 2018a; Uchida and Brown, 2004).

### Axon-to-axon variability

There was considerable variability in our measurements of the anterograde and retrograde rates, which is evident in the box-and-whisker plots. Some of this likely reflects statistical error introduced by the flat-field and photobleaching corrections. Additional variability likely arises from noise in our measurements because of the low expression level of paGFP-NFM (Walker et al., 2019). In the pulse-spread method, this problem is compounded by the infrequent movement of the filaments, which results in <1% of the fluorescence in the central window entering the flanking windows during the first 4 min after photoactivation.

In addition to these considerations, it is important to note that our pulse-spread method provides a snapshot in time at one location along each axon. This is very different from radioisotopic pulse-labeling studies, which represent the summation of thousands of axons on a timescale of weeks and over a distance of centimeters. The average axon diameter in our study at 8 weeks was 3 μm. Axons of this size contain ∼1000 neurofilaments per cross section (Reles and Friede, 1991). Assuming an average neurofilament length of 5–10 μm (based on measurements in cell culture; Wang and Brown, 2010; Fenn et al., 2018a) and a central window length of 40 μm, this means that on average the slopes in the flanking windows in our experiments may be based on the movement of only 20–40 neurofilaments (0.5% of 4000–8000 filaments). Since neurofilaments are likely to be longer *in vivo* than in cell culture (Burton and Wentz, 1992), this number is probably an overestimate. Given such small numbers and the stochastic nature of neurofilament movement, some of the scatter in our measurements may also be sampling error that would average out on longer timescales.

### Why is neurofilament transport bidirectional?

Axonal neurofilaments accumulate in high numbers during axonal growth and maturation, and this drives the expansion of axon caliber (Hoffman, 1995). The accumulation is possible because of the mechanisms of axonal transport, which deliver these polymers to the axon from their site of assembly in the cell body. However, if delivering neurofilaments to axons is the sole function of neurofilament transport, then why do neurons also invest energy to move these polymers backward? The fact that a significant fraction of axonal neurofilaments move retrogradely suggests that neurofilament movement must have another purpose.

Neurofilaments are highly flexible polymers with persistence lengths on the order of several hundred nanometers (Beck et al., 2010). However, electron micrographs of axons reveal a highly organized cytoskeletal arrangement in which the neurofilaments are fully extended and aligned in parallel to the long axis of the axon, forming an anisotropic array that can be likened to a loose bundle of cables (Hirokawa, 1982; Schnapp and Reese, 1982). A large myelinated axon can contain hundreds of thousands of neurofilament polymers that form a longitudinal overlapping array that extends the entire length of the axon. These polymers must also be distributed evenly throughout each successive internode to ensure a uniform axonal diameter from one internode to the next.

This remarkable organization of axonal neurofilaments raises two important questions. First, why are the neurofilaments aligned longitudinally? We suggest that this alignment is important for the axonal transport of other cargoes. To appreciate this, consider the extraordinary volume of membrane traffic in axons. Dozens of distinct classes of membranous organelles including Golgi-derived vesicles, endosomes, lysosomes, autophagosomes, mitochondria and peroxisomes are constantly being transported bidirectionally along microtubule tracks. As these polymers push through the axon, they must part the neurofilaments without entanglement. We speculate that the longitudinal alignment of neurofilaments is important because it allows them to fulfill their space-filling function in axons while also providing minimal resistance to the movement of these membranous cargoes.

A second important question is, how is the longitudinal organization and uniform distribution of neurofilaments along axons established and maintained? We speculate that this organization arises because of the action of opposing motors on neurofilaments, which function not just to transport these flexible polymers, but also to orient and distribute them longitudinally. Some support for this hypothesis comes from our live-imaging studies, which have revealed that neurofilaments often fold back on themselves in complex ways, but whenever they move, they tend to unfurl into a fully extended configuration, sometimes shuttling forward and backward as if being subjected to a tug-of-war between opposing motors (Taylor et al., 2012; Fenn et al., 2018b). We only rarely observed a filament move while folded. These observations suggest that the mechanisms of bidirectional neurofilament transport tend to stretch neurofilaments out both proximally and distally along the long axis of the axon, perhaps providing a mechanism by which the longitudinal organization and alignment of these polymers is established and maintained. Thus, the neurofilament cytoskeleton could be considered to be sculpted by motile forces, and the bidirectional movement of these polymers may function not just to deliver them to axons, but also to organize and distribute them.

### Neurofilament transport slows with age and distance along the nerve

Radioisotopic pulse-labeling studies in sciatic nerves of 7- to 8-week-old mice have demonstrated that the neurofilament transport velocity slows both spatially and temporally, from ∼0.6 mm/d 1–3 weeks after injection, when the wave is in the ventral root and proximal sciatic nerve, to ∼0.1 mm/d 4–6 weeks after isotope injection, when the wave has moved more distally (Xu and Tung, 2001; Jung and Brown, 2009). A similar spatial and temporal slowing has been reported in rat sciatic nerve (Hoffman et al., 1983, 1985; Watson et al., 1989).

The pulse-spread method has allowed us to make the first estimates of average neurofilament transport velocity *in vivo* by a method that is independent of radioisotopic pulse labeling. For 8-week-old mice, we estimate velocities of ∼0.04 mm/d in the sciatic nerve, declining to ∼0.02 mm/d in the tibial nerve, measured at locations ∼30 mm apart. This slowing was driven by a reduction in the anterograde transport of neurofilaments, while the retrograde transport remained unchanged. In addition, in the tibial nerve we found that the overall neurofilament population velocity decreased with age from an average velocity of ∼0.1 mm/d at 2 weeks to ∼0.02 mm/d at 16 weeks. Thus, our data confirm that there is both a spatial and temporal slowing of neurofilament transport in the sciatic and tibial nerves, and they indicate that the balance of anterograde and retrograde transport flux may be an important determinant of neurofilament transport velocity. Our modeling suggests that this most likely reflects an increase in the time that the filaments spend pausing (Jung and Brown, 2009). The temporal slowing correlated with an increase in average axon caliber, from 2.2 μm at 2 weeks to 4.1 μm at 16 weeks. Since a slowing of neurofilament transport results in an increase in the residence time of these polymers in the axon, it may contribute to the accumulation of neurofilaments that is necessary to drive this axonal expansion (Hoffman, 1995).

There are no published measurements of neurofilament transport velocity in the tibial nerve, but there are such data for the sciatic nerve. Our estimate of the velocity in the sciatic nerve was approximately 2-fold to 10-fold slower than the estimates obtained by radioisotopic pulse labeling (compare the ∼0.1–0.6 mm/d obtained by radioisotopic pulse labeling in mice injected at 7–8 weeks to ∼0.04 mm/d obtained with the pulse-spread technique at 8 weeks; Xu and Tung, 2001). However, the spatial and temporal scales of the fluorescence photoactivation pulse-spread and radioisotopic pulse-labeling methods are so different and the relative contributions of spatial and temporal factors to the slowing in the radioisotopic pulse-labeling studies are not known. Thus, it is not clear how to compare data obtained with these different approaches. In addition, it is important to note that our measurements were made exclusively on myelinated axons, whereas the radioisotopic pulse-labeling method represents an average of all axons in the nerve. Also, it is possible that our modeling may have undercorrected for the departure of fluorescent neurofilaments from the flanking windows during the period of measurement. As we noted in the Results, this can be a problem when the velocity or amount of neurofilament transport is high, such as in proximal axons and in younger animals. In fact, since the calculated velocity is proportional to the difference between the transport rates measured in the two flanking windows, we showed that small differences in those rates can lead to large differences in the estimated velocity.

### Conclusion

To summarize, we have described a fluorescence photoactivation paradigm that allows us to quantify the anterograde and retrograde movement of a pulse of fluorescent neurofilaments in mature myelinated axons of mouse sciatic nerve. Our data confirm that neurofilament transport has an anterograde bias and show that ∼40% of the filaments move retrogradely. This suggests that neurofilament transport has additional functions beyond simply delivering these polymers to axons. We speculate that one of these functions may be to distribute and align neurofilaments in axons, thereby allowing these space-filling polymers to fulfill their role without impeding the movement of other axonally transported cargoes.

## Synthesis

Reviewing Editor: Eran Perlson, Tel Aviv University Decisions are customarily a result of the Reviewing Editor and the peer reviewers coming together and discussing their recommendations until a consensus is reached. When revisions are invited, a fact-based synthesis statement explaining their decision and outlining what is needed to prepare a revision will be listed below. The following reviewer(s) agreed to reveal their identity: Peter Baas. Note: If this manuscript was transferred from JNeurosci and a decision was made to accept the manuscript without peer review, a brief statement to this effect will instead be what is listed below.

This is a beautiful and important work demonstrating neurofilament transport in vivo for the first time. Neurofilament transport has been visualized in cell culture but not in vivo. This paper does so, which is a significant contribution especially given that there may have been some doubt regarding whether the bidirectional transport of neurofilaments in cultured neurons reflects neurofilament behavior in vivo, in adult neurons, and aging neurons.

The authors should address the following issues:

1. The effects the investigators are quantifying are challenging to see in both the figures and the movies. The investigators should create one more figure (and/or movie) in which some kind of pseudocolor or arrows or any other special effects could help the observer see what the software is detecting and quantifying. For many readers, it would make all the difference in the world.

2. I doubt the n defined for specific experiments (3D-F; 4B-D). In these cases, the n corresponds to each individual axon. This is probably pseudo-replication, and alternatively, each individual animal should be considered as the experimental n. The authors should provide this analysis.

3. Given that the values predicted by the model show a high degree of variation when compared to experimental data (except in the case of the % of anterograde transport), what is its real applicability?

4. Discussing the putative functions of bidirectional transport of neurofilaments should be expanded.
